# *Desulfovulcanus ferrireducens* gen. nov., sp. nov., a thermophilic autotrophic iron and sulfate-reducing bacterium from subseafloor basalt that grows on akaganéite and lepidocrocite minerals

**DOI:** 10.1007/s00792-022-01263-2

**Published:** 2022-02-21

**Authors:** Srishti Kashyap, Masroque Musa, Kaylee A. Neat, Deborah A. Leopo, James F. Holden

**Affiliations:** 1 Department of Microbiology, University of Massachusetts, N418 Morrill IV North; 639 N. Pleasant St., Amherst, MA 01003 USA; 2 Department of Astronomy, Mount Holyoke College, South Hadley, MA 01075 USA; 3 Present Address: Department of Geological Sciences, University of Colorado, Boulder, CO 80309 USA

**Keywords:** Deep sea thermophiles, Anaerobic bacteria, Autotroph, Iron reducer, Sulfate reducer, Hydrothermal vent

## Abstract

A deep-sea thermophilic bacterium, strain Ax17^T^, was isolated from 25 °C hydrothermal fluid at Axial Seamount. It was obligately anaerobic and autotrophic, oxidized molecular hydrogen and formate, and reduced synthetic nanophase Fe(III) (oxyhydr)oxide minerals, sulfate, sulfite, thiosulfate, and elemental sulfur for growth. It produced up to 20 mM Fe^2+^ when grown on ferrihydrite but < 5 mM Fe^2+^ when grown on akaganéite, lepidocrocite, hematite, and goethite. It was a straight to curved rod that grew at temperatures ranging from 35 to 70 °C (optimum 65 °C) and a minimum doubling time of 7.1 h, in the presence of 1.5–6% NaCl (optimum 3%) and pH 5–9 (optimum 8.0). Phylogenetic analysis based on 16S rRNA gene sequences indicated that the strain was 90–92% identical to other genera of the family *Desulfonauticaceae* in the phylum *Pseudomonadota*. The genome of Ax17^T^ was sequenced, which yielded 2,585,834 bp and contained 2407 protein-coding sequences. Based on overall genome relatedness index analyses and its unique phenotypic characteristics, strain Ax17^T^ is suggested to represent a novel genus and species, for which the name *Desulfovulcanus ferrireducens* is proposed. The type strain is Ax17^T^ (= DSM 111878^T^ = ATCC TSD-233^T^).

## Introduction

Most thermophilic chemoautotrophs studied from deep-sea hydrothermal vents are methanogens and other microbes that oxidize molecular hydrogen (H_2_) and reduce sulfur compounds and nitrate (Sievert and Vetriani 2012). Less is known about thermophilic iron reducers that use molecular hydrogen as an electron donor and Fe(III) (oxyhydr)oxide minerals as terminal electron acceptors. These include the facultatively autotrophic bacteria *Deferribacter abyssi* and *Deferribacter autotrophicus* with optimal growth at 60 °C (Miroshnichenko et al. 2003; Slobodkina et al. 2009a), the facultatively autotrophic archaea *Geoglobus ahangari* and *Geoglobus acetivorans* with optimal growth at 81–88 °C (Kashefi et al. 2002; Slobodkina et al. 2009b), and the chemolithoheterotrophic archaeon *Pyrodictium delaneyi* Su06^T^ with optimal growth at 90–92 °C (Lin et al. 2014, 2016; Kashyap and Holden 2021).

Ferrihydrite (Fe_2_O_3_∙0.5H_2_O) is the most common Fe(III) (oxyhydr)oxide mineral used for the growth of iron-reducing bacteria and archaea. It is the least crystalline iron oxide in a gradient that includes (from least to most crystalline) lepidocrocite (γ-FeOOH), akaganéite (β-FeOOH), maghemite (γ-Fe_2_O_3_), goethite (α-FeOOH), and hematite (α-Fe_2_O_3_), which makes ferrihydrite a more favorable electron acceptor (Cornell and Schwertmann 2003). Furthermore, nanophase iron oxide minerals (< 100 nm in at least one dimension) are also more reactive than their macroparticulate counterparts due to an increase in reactive surface area (Braunschweig et al. 2013). When tested on various synthetic nanophase Fe(III) (oxyhydr)oxides, *P. delaneyi* grew best and produced the most acid soluble Fe^2+^ on ferrihydrite and showed modest growth and Fe^2+^ production on lepidocrocite and akaganéite with poor growth and Fe^2+^ production on goethite and hematite (Kashyap et al. 2018). It reduced ferrihydrite to magnetite (Fe_3_O_4_) (Lin et al. 2014; Kashyap et al. 2018), lepidocrocite to a ferrous carbonate mineral, and akaganéite to a ferrous phosphate mineral and magnetite (S. Kashyap and J. Holden, unpubl. results).

This study sought to isolate and characterize a thermophilic, obligate autotroph that uses synthetic nanophase akaganéite and lepidocrocite as terminal electron acceptors from low-temperature hydrothermal fluid flowing from a basalt outcrop at Axial Seamount. The goal was to determine if there are chemoautotrophic bacteria that are better adapted for growth on either nanophase akaganéite or lepidocrocite rather than nanophase ferrihydrite. A novel genus and species, *Desulfovulcanus ferrireducens* Ax17^T^, in the family *Desulfonauticaceae* (Waite et al. 2020) that grew as well or better on akaganéite and lepidocrocite relative to ferrihydrite was isolated and characterized and its whole genome was sequenced to determine its novelty and metabolic potential.

## Materials and methods

### Isolation of new thermophile strains

Hydrothermal vent samples were collected in July 2017 from Axial Seamount in the northeastern Pacific Ocean (45.9° N, 130.0° W) on board the R/V *Roger Revelle* using the remotely operated submarine ROV *Jason* II at a depth of 1513 m. Low-temperature (25 °C) hydrothermal vent fluid was collected from Marker 33 vent using the NOAA Hydrothermal Fluid and Particle Sampler (Butterfield et al. 2004). The hydrothermal fluid was used to inoculate growth media (see below) that separately contained akaganéite and lepidocrocite as terminal electron acceptors and 80% (v/v) H_2_ and 20% (v/v) CO_2_ at 2 atm in the headspace as the carbon source and electron donor. The enrichments were incubated at sea at 55 °C in a forced-air incubator for up to 7 days.

Enrichments were screened for cell growth using epifluorescence microscopy, Fe^2+^ production using a spectrophotometer (see below), and CH_4_ production in the headspace using a gas chromatograph. Enrichments that showed cell growth and evidence of iron reduction without production of CH_4_ were transferred at least three additional times to confirm growth. Two enrichments from Marker 33 hydrothermal vent fluid that grew at 55 °C using akaganéite and lepidocrocite as electron acceptors were separately isolated on their respective iron oxides using at least three sequential dilution-to-extinction incubations where the most dilute sample in the series to show growth was used to inoculate the next dilution series or, for the final dilution series, was used as the purified strain. 16S rRNA gene sequence analysis of the two isolates showed that they had identical sequences. Therefore, only the akaganéite-grown strain was used for further characterization and was named Ax17^T^.

### Growth conditions

The growth medium for all laboratory experiments, except where amended (see below) was based on DSM Medium 981 (Kashefi et al. 2002) that contained (per liter) 19.0 g of NaCl, 9.0 g of MgCl_2_∙6H_2_O, 0.30 g of CaCl_2_∙2H_2_O, 0.50 g of KCl, 0.42 g of KH_2_PO_4_, 0.05 g of NaBr, 0.02 g of SrCl_2_∙6H_2_O, 0.15 g of MgSO_4_∙7H_2_O, 0.1 g of (NH_4_)_2_SO_4_, 1.0 g of NaHCO_3_, 0.16 g of FeCl_2_ (for iron grown cultures), 10 ml of DSM Medium 141 trace element solution, and 10 ml of DSM Medium 141 vitamin solution. In the absence of iron as a terminal electron acceptor (see below), 50 μl of a 0.5% (w/v) resazurin solution was added as a redox indicator. The medium was pH balanced to 6.80 ± 0.05 (room temperature) and 0.5 mM cysteine-HCl was added prior to inoculation as the reducing agent. Cultures were grown in sealed Balch tubes and serum bottles sealed with butyl rubber stoppers and 80% (v/v) H_2_ and 20% (v/v) CO_2_ at 2 atm in the headspace in a forced-air incubator. Strain Ax17^T^ was incubated at 55 °C unless otherwise stated.

Nanophase akaganéite, lepidocrocite, ferrihydrite, goethite, and hematite (each 100 mmol Fe(III) (oxyhydr)oxide per liter) were separately tested as terminal electron acceptors. They were synthesized and kept at 4 °C in the dark in concentrated aqueous solutions as previously described (Sklute et al. 2018). Other terminal electron acceptors tested were 20 mM Fe(III)-citrate, 7.0 mM sodium sulfate, 7.9 mM sodium sulfite, 6.3 mM sodium thiosulfate, 1% (w/v) elemental sulfur (equivalent to 313 mmol total sulfur per liter or 40 mmol cyclooctasulfur per liter), 9.9 mM potassium nitrate, 40% (v/v) H_2_ and 10% (v/v) CO_2_ in air at 2 atm, and 100% air at 2 atm. 80% (v/v) H_2_ and 20% (v/v) CO_2_ at 2 atm, 10 mM each of D-glucose, D-maltose, L-alanine, ethanol, glycerol, and sodium salts of formate, acetate, pyruvate, citrate, and succinate; 0.5% (w/v) tryptone (pancreatic digest of casein, Oxoid Ltd.); and 0.02% (w/v) Bacto yeast extract (Becton, Dickinson and Company) were tested separately as carbon and electron donors using 80% (v/v) N_2_ and 20% (v/v) CO_2_ at 2 atm in the headspace (except for the H_2_:CO_2_ condition).

For kinetic experiments, strain Ax17^T^ was grown in triplicate on ferrihydrite at temperatures ranging from 30 to 75 °C; at pH 4 (no buffer), pH 5 and 6 (5 mM MES buffer) pH 7 and 8 (20 mM PIPES buffer), and pH 8 and 9 (100 mM EPPS buffer); and 0.11 M to 1.26 M chloride to determine their effect on growth. A pH above pH 9 was not sustainable with incubation, and therefore not tested. Strain Ax17^T^ was also grown in duplicate on akaganéite, lepidocrocite, ferrihydrite, goethite, hematite, sodium sulfate, elemental sulfur, and control medium without an added electron acceptor. Each of these was matched with an uninoculated control. At various time points, an aliquot from each bottle was preserved with 2% formaldehyde (v/v) and mixed 1:1 in a filter-sterilized anoxic oxalate solution (0.23 M ammonium oxalate-0.17 M oxalic acid, Phillips and Lovley 1987) to dissolve the iron oxide minerals (when present). The concentration of cells in each bottle was determined by epifluorescence microscopy (Hobbie et al. 1977). Cells were filtered onto a 0.2-µm-pore-size membrane filter pre-stained with Irgalan black (Whatman), stained with 0.1% (w/v) acridine orange for 2 min, and counted with a Nikon Eclipse E400 microscope. The specific growth rate of the culture was determined by a best-fit curve to the logarithmic portion of the growth data. Total oxalate fixed ferrous iron and Zn fixed sulfide concentrations were determined spectrophotometrically using the ferrozine assay (Phillips and Lovley 1987) and the methylene blue assay (Chen and Mortenson 1977), respectively. Confidence intervals (95%) were calculated for all specific growth rates and an ANCOVA and Tukey tests (*α* = 0.05) were run on specific growth rates for the varying terminal electron acceptors as described previously (Zar 1996).

### Electron microscopy

For negative staining of whole mounted cells, 10 ml of culture within a sealed Balch tube were fixed by adding 0.2 ml of 50% (v/v) electron microscopy-grade glutaraldehyde with gentle mixing and incubating at room temperature for 1 h. An aliquot (3 ml) of the fixed culture was then removed from the sealed Balch tube and applied to plasma-treated carbon films (ca. 0.5 nm thickness) on 400 mesh copper grids. The grids were stained with 3% (w/v) NH_4_OH and 2% (w/v) aqueous uranyl acetate and viewed with a JEOL-100S transmission electron microscope.

### 16S rRNA gene and genome sequence analyses

The genomic DNA of strain Ax17^T^ was extracted and purified using a Wizard genomic DNA purification kit (Promega, USA) per the manufacturer’s protocol. The 16S rRNA gene was amplified using the polymerase chain reaction (PCR) and sequenced. The bacterial primers used were 27f-CM (5′-AGAGTTTGATCMTGGCTCAG-3′, Frank et al. 2008) and 1492r (5′-GGTTACCTTGTTACGACTT-3′, Wilson et al. 1990) as one PCR pair and 338f (5′-ACTCCTACGGGAGGCAGC-3′, Whitely and Bailey 2000) and 1391r (5′-GACGGGCRGTGWGTRCA-3′, Brunk and Eis 1998) as another pair (Integrated DNA Technologies). Each PCR reaction contained 21.5 μl of nuclease-free water (Fisher BioReagents), 3 μl of 10 × Omni Klentaq mutant reaction buffer (DNA Polymerase Technology), 2.4 μl of 2.5 μM dNTPs (Promega), 1 μl each of 10 μM forward and reverse primers, 0.1 μl of Omni Klentaq DNA polymerase (DNA Polymerase Technology, Inc.), and 1 μl of DNA template. The PCR consisted of 96 °C for 5 min followed by 30 cycles of 95° for 30 s, 54 °C for 30 s, and 68 °C for 30 s, then 68 °C for 5 min followed by 25 °C for 1 min. The PCR products were cleaned using a DNA Clean & Concentrator Kit (Zymo Research) and sequenced using Sanger sequencing in the forward and reverse directions (Genewiz, Inc.). A consensus sequence was derived from the combined sequences. Phylogenetic trees were constructed using 16S rRNA gene sequences from NCBI using the Maximum Likelihood method and Tamura-Nei model (Tamura and Nei 1993). Evolutionary analyses were conducted in MEGA X (Kumar et al. 2018). The 16S rRNA gene sequence for Ax17^T^ was deposited at DDBJ/ENA/GenBank under the accession number MZ048018.

Following genomic DNA extraction as described above, library construction for whole genome sequencing was performed using a NexteraXT DNA library prep kit (Illumina, USA) per the manufacturer’s protocol. Both library construction and sequencing were performed by Genewiz, Inc. (South Plainfield, NJ, USA). The DNA was sequenced using a MiSeq instrument (Illumina, USA) with 2 × 150-bp chemistry. Default parameters were used for all software analyses. Trimmomatic version 0.36 (Bolger et al. 2014) was used to trim the last 8 bp of each sequence and regions with low-quality Q scores (Q < 30). The resulting paired-end sequences were then assembled using SPAdes genome assembler version 3.10 (Nurk et al. 2013). The completeness of the genome sequence was determined using CheckM (Park et al. 2015). Open reading frames (ORFs) were identified using EMBOSS tools (Rice et al. 2000) and annotated using Diamond BLASTp (Buchfink et al. 2015). Hydrogenases were classified using HydDB (Søndergaard et al. 2016) and Type IV pili proteins were identified using PilFind version 1.0 (Imam et al. 2011). rRNA genes were identified using RNAmmer version 1.2 (Lagesen et al. 2007) and tRNA genes were identified using tRNAscan-SE version 2.0 (Lowe and Chan 2016). This whole genome shotgun sequencing project was deposited at DDBJ/ENA/GenBank under the accession number JAGUQP000000000. The version described in this paper is version JAGUQP010000000. The raw reads were deposited in the Sequence Read Archive under run number SUB8811277 and BioProject number PRJNA688464.

For overall genome relatedness index (OGRI) analyses, the BLAST-based average nucleotide identity (ANI) and Alignment Fraction (AF) scores were calculated using the JSpeciesWS program, version 3.2.2 (Richter et al. 2016). Genome-to-genome direct comparison (GGDC) analyses were performed using Eq. 2 in the GGDC program, version 2.1 (Meier-Koltoff et al. 2013). Default parameters were used for all software analyses. Forty marker proteins defined for the Species Identification (SpecI) analyses (Mende et al. 2013) were compared using BLAST-P. Ribosomal multilocus sequence typing (rMLST) analyses (Jolley et al. 2012) were performed by trimming and concatenating 50 ribosomal protein sequences from 10 whole genome sequences in NCBI and aligning them using the Maximum Likelihood method and a Jones-Taylor-Thorton matrix-based model (Jones et al. 1992). Evolutionary analyses were conducted in MEGA X (Kumar et al. 2018).

## Results and discussion

### Phenotypic characteristics

**Fig. 1:**
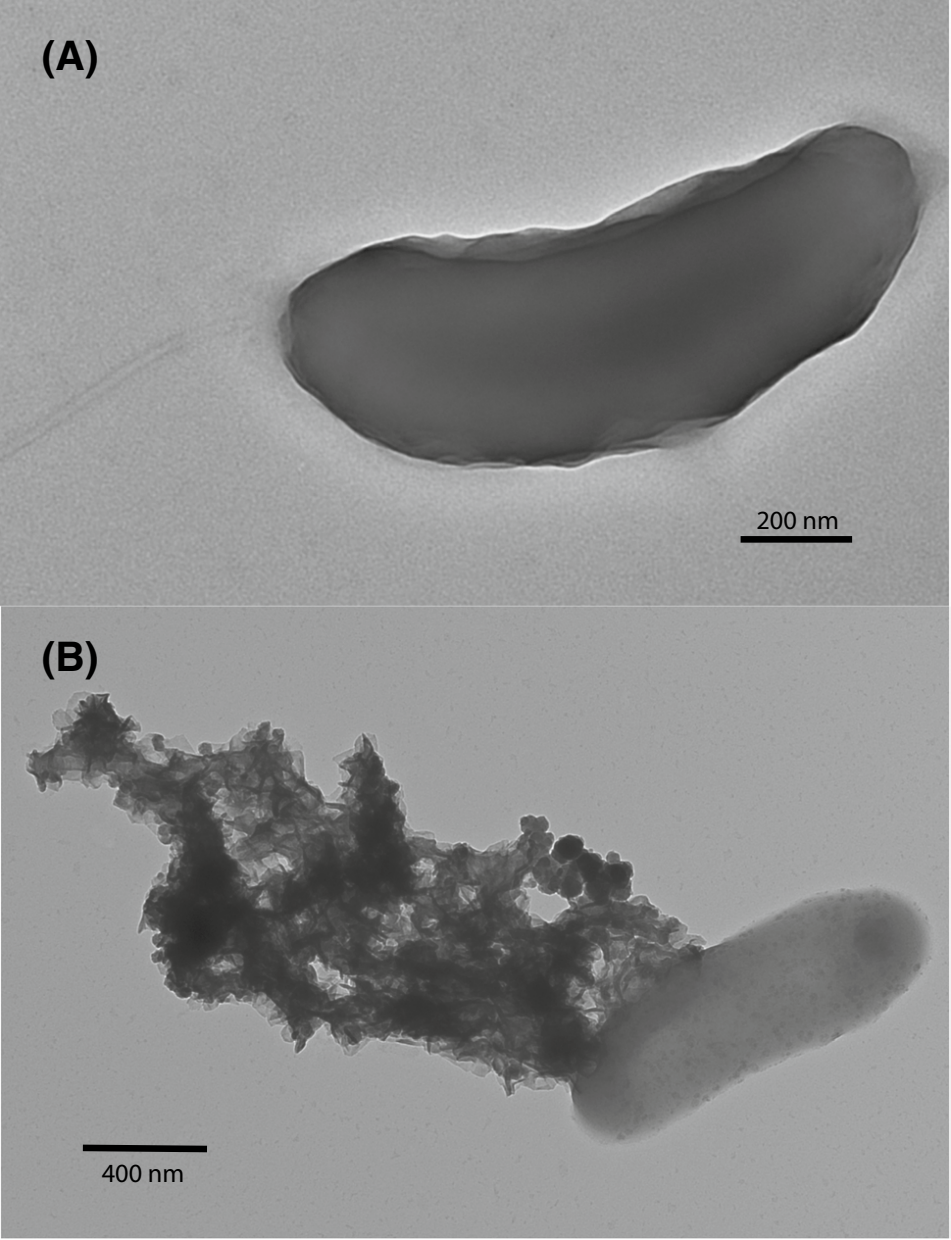
Transmission electron micrograph of strain Ax17^T^ showing **a** a rod-shaped cell with a monopolar flagellum and **b** a rod-shaped cell with transformed akaganéite following growth on the mineral

**Fig. 2:**
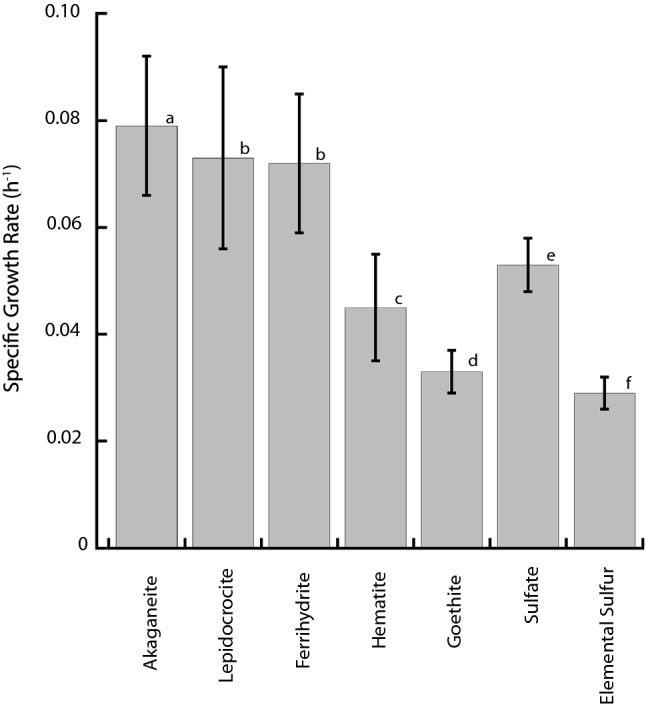
Specific growth rates for strain Ax17^T^ when grown on varying terminal electron acceptors. The error bars represent 95% confidence intervals. The letters represent statistically different groups of rates (*p* < 0.05) following an analysis of covariance and a Tukey test

Strain Ax17^T^ is an obligately anaerobic, thermophilic autotroph. Electron microscopy revealed straight to curved rods, 1.5 µm by 0.4 µm, with monopolar flagellation (Fig. 1). It grew on akaganéite, lepidocrocite, ferrihydrite, hematite, goethite, sulfate, sulfite, thiosulfate, and elemental sulfur as terminal electron acceptors (Table 1). At its optimal growth temperature, the specific growth rate of strain Ax17^T^ was highest when grown on akaganéite (0.079 h^−1^ ± 0.013 h^−1^, 95% confidence interval) followed by growth on lepidocrocite (0.073 h^−1^ ± 0.017 h^−1^) and ferrihydrite (0.072 h^−1^ ± 0.013 h^−1^), which were not significantly different (Fig. 2). Specific growth rates were lower when hematite (0.045 h^−1^ ± 0.010 h^−1^), goethite (0.033 h^−1^ ± 0.004 h^−1^), sulfate (0.053 h^−1^ ± 0.005 h^−1^), and elemental sulfur (0.029 h^−1^ ± 0.003 h^−1^) were used as terminal electron acceptors (Fig. 2). Strain Ax17^T^ produced up to 20 mM Fe^2+^ when grown on ferrihydrite relative to abiotic controls but < 5 mM Fe^2+^ when grown on akaganéite, lepidocrocite, hematite, and goethite. Strain Ax17^T^ did not grow when Fe(III)-citrate, nitrate, or oxygen was supplied as the electron acceptor nor in the absence of an added electron acceptor.

**Table 1:** Differential characteristics of Ax17^T^ and members of the families *Desulfonauticaceae* and *Desulfonotronovibrionaceae*

Characteristics^a^	Ax17^T^	*Desulfonauticus submarinus* 6N^T^	*Desulfonauticus autotrophicus* TeSt^T^	*Desulfonatronovibrio hydrogenovorans* Z-7935^T^	*Desulfonatronospira thiodismutans* ASO3-1^T^
Habitat	Hydrothermal vent, Axial Seamount, Pacific Ocean	Hydrothermal vent, 13° N East Pacific Rise, Pacific Ocean	Oil reservoir, Hamburg, Germany	Lake Magadi, Kenya	Kulanda Steppe, Russia
16S rRNA gene identity (%)	100	90.61	90.68	90.95	86.69
OGRI analyses					
ANI (%)	100	67.22	ND	66.66	65.93
AF	100	0.2943	ND	0.2922	0.2480
GGDC (%)	100	21.0	ND	19.6	23.2
SpecI (%)	100	77.4	ND	76.2	74.2
rMLST (%)	100	77.7	ND	75.6	ND
Morphology					
Cell shape	Straight to curved rods	Curved rods	Straight to curved rods	Vibrio	Vibrio to spirillum
Length (μm)	1.5	5–6	1.2–4	1.5–2	2–30
Width (μm)	0.4	0.4–0.5	0.6	0.5	0.6–0.8
Flagellation	Monopolar	Monopolar	Monopolar	Monopolar	Monopolar
Temperature range of growth and (optimum) (°C)	35–70 (65)	30–60 (45)	40–64 (58)	22–40 (37)	up to 43
pH range of growth and (optimum)	5.0–9.0 (8.0)	(7.0)	(7.0)	8–10.2 (9.5–9.7)	8.3–10.5 (10)
NaCl range of growth and (optimum) (%)	1.5–6 (3)	0–5 (2)	1–6 (3)	1–12 (3)	8.7–23.2
Minimum doubling time	7.1 h	ND	80 min	ND	ND
Electron donors					
H_2_	+	+ ^b^	+ ^b^	+ ^bc^	+
Formate	+	+ ^b^	+ ^b^	+ ^bc^	+
Acetate	–	–	–	–	+
Pyruvate	–	ND	–	–	+
Glucose	–	–	–	–	ND
Electron acceptors					
Fe(III) oxides^d^	+	ND	ND	ND	ND
Fe(III) citrate	–	ND	–	ND	ND
Sulfate	+	+	+	+	+
Sulfite	+	+	+	+	+
Thiosulfate	+	+	+	+	+
Elemental sulfur	+	+	+	–	–
Nitrate	–	–	–	–	ND
Oxygen	–	–	–	–	–

+ Positive; – negative; *ND* not determined

^a^Data from Zhilina et al. 1997; Audiffin et al. 2003; Sorokin et al. 2008; Mayilraj et al. 2009

^b^Growth on H_2_ + acetate or formate + acetate

^c^Yeast extract requirement for growth

^d^Ferrihydrite, lepidocrocite, akagenéite, goethite, and hematite, each tested separately

**Fig. 3:**
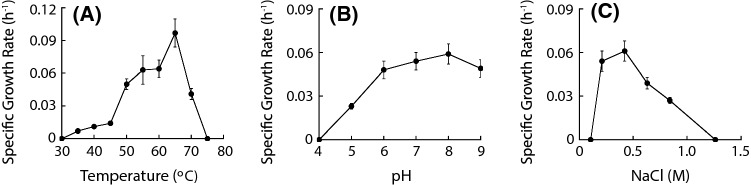
Specific growth rates for strain Ax17^T^ across its growth range of **a** temperature, **b** pH, and **c** chlorinity. The error bars represent 95% confidence intervals

Strain Ax17^T^ only used molecular hydrogen and formate separately as electron donors and did not utilize acetate, glucose, tryptone, pyruvate, citrate, succinate, ethanol, glycerol, or yeast extract as an alternative source of carbon and electrons (Table 1). Growth was observed on molecular hydrogen and ferrihydrite between 35 and 70 °C with an optimum of 65 °C (Fig. 3a), between pH 5.0 and 9.0 with an optimum of pH 8.0 (Fig. 3b), and between 0.21 M and 0.84 M Cl^−^ with an optimum of 0.42 M Cl^−^ (Fig. 3c). Its minimum doubling time was 7.1 h.

### 16S rRNA phylogenetic analysis

**Fig. 4:**
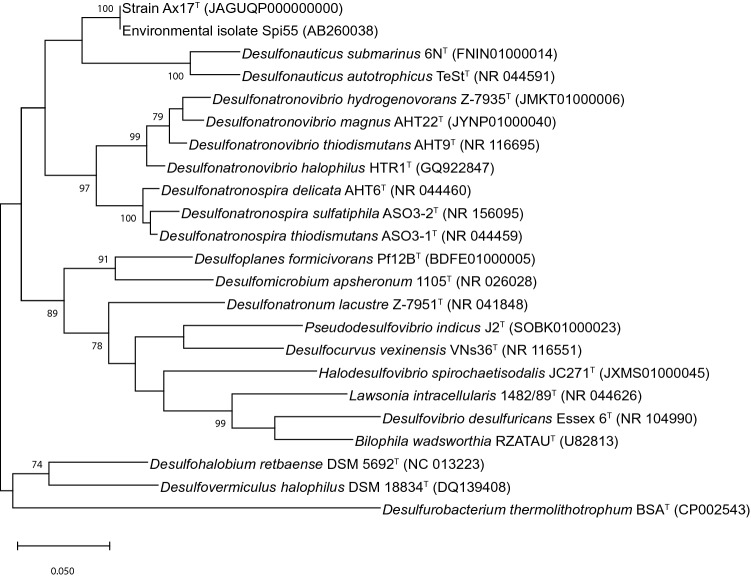
Phylogenetic tree based on the 16S rRNA gene showing the position of Ax17^T^ within the order *Desulfovibrionales* including at a minimum the type strain of every genus. After 1000 bootstrap constructions, the tree with the highest log likelihood (− 10,800) is shown, with values next to nodes indicating the percentage of reconstructions in which the topology was preserved (values < 70% are omitted for clarity). There were a total of 1472 nt positions in the final dataset. Branch lengths are to scale and indicate the number of substitutions per site; bar, 5 substitutions per site. The outgroup is *Desulfurobacterium thermolithotrophum* BSA^T^ from the phylum *Aquificota* (formerly *Aquificae*). GenBank/EMBL/DDBJ accession numbers are included in parentheses

The 16S rRNA sequence obtained from the whole genome sequence was nearly identical (1427 of 1429 nucleotides) to the consensus sequence obtained by PCR and was used as the final 16S rRNA sequence for the organism. Based on its 16S rRNA gene sequence, strain Ax17^T^ was most closely related to members of the family *Desulfonauticaceae* (Waite et al. 2020) (Fig. 4) in the phylum *Pseudomonadota* (Oren and Garrity 2021). It showed highest sequence identities to *Desulfonauticus autotrophicus* TeSt^T^ (90.68%), *Desulfonauticus submarinus* 6N^T^ (90.61%), *Desulfonatronovibrio hydrogenovorans* Z-7935^T^ (90.95%), and *Desulfonatronospira thiodismutans* ASO3-1^T^ (86.69%) (Table 1). Each of these sequence identities were below the 98.7% identity demarcation for a novel species (Chun et al. 2018), the 94.5% identity demarcation for a novel genus (Yarza et al. 2014), and the 92.25% identity for family but above the order demarcation (89.20% identity) using the ‘Yarza medians’ (Boden et al. 2017).

### Genome sequence analysis

Whole genome sequencing using a MiSeq instrument generated a total of 18,566,594 raw paired-end reads and 5,570 Mb of sequenced bases. Genome assembly resulted in 60 high-quality contigs, with an *N*_50_ value of 77,684 bp and a maximum contig length of 262,489 bp. The assembled Ax17^T^ genome was 2,585,834 bp long and 98.2% complete with > 2,000-fold average coverage resulting in 2,407 protein-coding genes and a G + C content of 42.5%. One copy each of the 5S, 16S, and 23S rRNA genes, and 60 tRNA genes were identified.

**Fig. 5:**
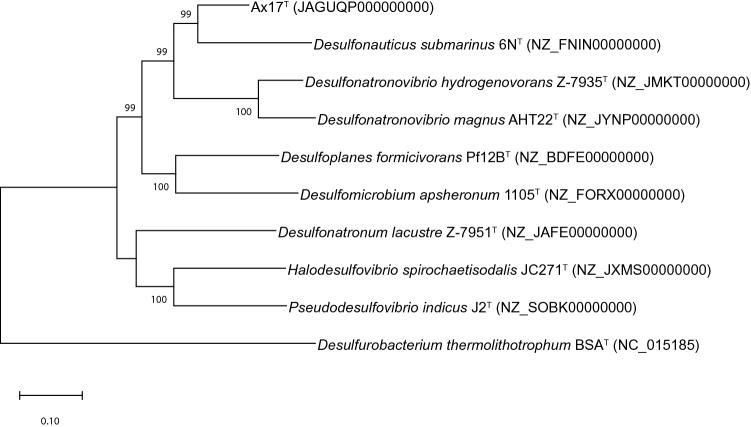
Phylogenetic tree of strain Ax17^T^ as inferred using ribosomal multilocus sequence typing (rMLST) of 50 concatenated ribosomal protein amino acid sequences. The tree with the highest log likelihood (-87,485) is shown. The percentage of trees in which the associated taxa clustered together is shown next to the branches (> 70%). The tree is drawn to scale, with branch lengths measured in the number of substitutions per site. There was a total of 7,340 positions in the final dataset. The outgroup is *Desulfurobacterium thermolithotrophum* BSA^T^ from the phylum *Aquificota* (formerly *Aquificae*). GenBank/EMBL/DDBJ accession numbers are included in parentheses

OGRI analyses also showed that strain Ax17^T^ was generally most closely related to *D. submarinus* 6N^T^ (Table 1). The ANI and AF scores for strain comparisons between Ax17^T^ and *D. submarinus* 6N^T^ were 67.2% and 0.2943, between Ax17^T^ and *D. hydrogenovorans* Z-7935^T^ were 66.7% and 0.2922, and between Ax17^T^ and *D. thiodismutans* ASO3-1^T^ were 65.9% and 0.2480, which were all below the ANI 96% cut-off and 0.6 AF values for species determination (Chun et al. 2018) and the ANI 73.1–74.0% cut-off value for genus determination (Barco et al. 2020) by this approach. The GGDC scores for strain comparisons between Ax17^T^ and *D. thiodismutans* ASO3-1^T^ was 23.2%, between Ax17^T^ and *D. submarinus* 6N^T^ was 21.0%, and between Ax17^T^ and *D. hydrogenovorans* Z-7935^T^ was 19.6% (Table 1), which were all below the 70% cut-off for delineating species by this approach (Chun et al. 2018). The SpecI-type protein analysis for strain Ax17^T^ gave values of 77.4% for *D. submarinus* 6N^T^, 76.2% for *D. hydrogenovorans* Z-7935^T^, and 74.2% for *D. thiodismutans* ASO3-1^T^ (Table 1), which are all below the 96.5% cut-off for delineating species by this approach (Mende et al. 2013). Therefore, all three OGRI analyses indicated that strain Ax17^T^ represents a novel genus and species. The rMLST phylogenetic tree confirmed that Ax17^T^ was most closely related to *D. submarinus* 6N^T^ (Fig. 5).

Like strain Ax17^T^, *D. submarinus*, *D. autotrophicus*, and *D. hydrogenovorans* only use molecular hydrogen and formate as electron donors, although the latter organisms must simultaneously use acetate as a carbon source and *D. hydrogenovorans* requires yeast extract for growth (Table 1) (Zhilina et al. 1997; Audiffin et al. 2003; Mayilraj et al. 2009). Only strain Ax17^T^ grew only on H_2_ and CO_2_ alone. Strain Ax17^T^ is also phenotypically similar to *D. submarinus* and *D. autotrophicus* based on its thermophilic optimal growth temperature and its ability to use sulfate, sulfite, thiosulfate, and elemental sulfur as electron acceptors (Table 1).

**Fig. 6:**
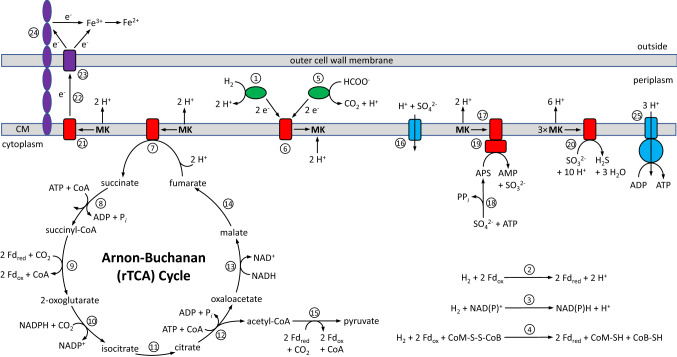
Proposed electron transport, energy generation, and CO_2_ fixation in strain Ax17^T^ based on bioinformatic analyses of its whole genome sequence. The putative enzymes are as follows: 1, periplasmic [NiFe] and [FeFe] cytochrome *c* hydrogenases (EC 1.12.2.1); 2, cytoplasmic ferredoxin (Fd)-reducing hydrogenase (EC 1.12.7.2); 3, cytoplasmic NAD(P)^+^-reducing hydrogenase (EC 1.12.1.5); 4, cytoplasmic H_2_:CoB-CoM heterodisulfide and ferredoxin reductase (EC 1.8.98.5); 5, periplasmic formate:cytochrome *c* dehydrogenase (EC 1.17.2.3); 6, membrane-bound menaquinone reductase (EC 7.1.1.8); 7, fumarate reductase (EC 1.3.5.4); 8, succinyl-CoA synthetase (EC 6.2.1.5); 9, 2-oxoglutarate:ferredoxin oxidoreductase (EC 1.2.7.3); 10, isocitrate dehydrogenase (EC 1.1.1.42); 11, aconitate hydratase (EC 4.2.1.3); 12, ATP citrate lyase (EC 2.3.3.8); 13, malate dehydrogenase (EC 1.1.1.37); 14, fumarate hydratase (EC4.2.1.2); 15, pyruvate:ferredoxin oxidoreductase (EC 1.2.7.1); 16, H^+^:SO_4_^2−^ symporter; 17, quinone-interacting membrane-bound oxidoreductase (EC 7.1.1.8), 18, sulfate adenylyltransferase (EC 2.7.7.4); 19, adenylylsulfate reductase (EC 1.8.99.2); 20, dissimilatory sulfite reductase (EC 1.8.99.5); 21, putative membrane *c*-type cytochrome; 22, putative periplasmic *c*-type cytochrome; 23, putative outer membrane *c*-type cytochrome; 24, putative e-pilin; and 25, membrane-bound ATP synthase (EC 7.1.2.2). *MK* menaquinone; *Fd* ferredoxin; *CoA* coenzyme A; *CoM*-*S–S-CoB* heterodisulfide; *CoM* reduced coenzyme M-SH; *CoB*-*SH* reduced coenzyme B

The Ax17^T^ draft genome contains putative genes that based on sequence identity at the amino acid level encode for a periplasmic [NiFe] cytochrome *c* hydrogenase (Group 1b, EC 1.12.2.1), a periplasmic [FeFe] cytochrome *c* hydrogenase (Group A1, EC 1.12.2.1), a cytoplasmic NAD(P)-reducing hydrogenase (Group A, EC 1.12.1.5), a cytoplasmic H_2_:CoB-CoM heterodisulfide and ferredoxin reductase (Group 3c, EC 1.8.98.5), and a cytoplasmic [FeFe] ferredoxin-reducing hydrogenase (Group B, EC 1.12.7.2) using the classification system of Greening et al. (2016). It has putative genes for two periplasmic formate:cytochrome *c* dehydrogenases (EC 1.17.2.3) that provide the organism with electrons and contributes to a proton motive force, a membrane-bound menaquinone reductase (QrcABCD, EC 7.1.1.8) that further contributes to a proton motive force, and a membrane-bound F-type ATP synthase (EC 7.1.2.2) that likely use the proton motive force for oxidative phosphorylation (Fig. 6).

The draft genome also contains putative genes that based on sequence identity at the amino acid level encodes for 9 monoheme *c*-type cytochromes and 10 *c*-type cytochromes with two or more hemes, based on the presence of CXXCH motifs and a signal peptide sequence (Gomi et al. 2004). The genome contains 12 type IV pilin-like signal peptides and their prepilin peptidase cleavage sites. One of these contains ≥ 9.8% aromatic amino acids with aromatic gaps of 23 amino acids or less, which suggests it is an electrically conductive pili (Walker et al. 2018; Bray et al. 2020), and another that shared 58% sequence identity with the type IV conductive pili from *Geobacter sulfurreducens*. The genome also contains putative genes for sulfate reduction: sulfate adenylyltransferase (Sat, EC 2.7.7.4), quinone-interacting membrane-bound oxidoreductase (QmoABC, EC 7.1.1.8), adenylylsulfate reductase (AprAB, EC 1.8.99.2), and dissimilatory sulfite reductase (DsrMKJOPABDC, EC 1.8.99.5) (Fig. 6). The genome contains putative genes that encode for all the proteins of the Arnon-Buchanan Cycle (i.e., the reductive TCA (rTCA) cycle), including ATP citrate lyase (EC 2.3.3.8), for CO_2_ fixation (Fig. 6).

Strain Ax17^T^ was isolated from low-temperature (25 °C) hydrothermal fluid emanating from a basalt outcrop at Marker 33 on Axial Seamount. Reactive transport modeling suggests there is considerable molecular hydrogen consumption by thermophiles at this and another low-temperature vent (Marker 113) at Axial Seamount (Stewart et al. 2019). Metagenomic analyses of vent fluids collected at these two sites showed that *Desulfonauticaceae* comprised 0.6–0.9% of the metagenomic reads of Marker 33 fluids and 1.7–1.9% of the reads of Marker 113 fluids (Fortunato et al. 2018). Strain Ax17^T^ showed 100% 16S rRNA gene sequence similarity with environmental isolate Spi55 from Ocean Drilling Program borehole 1026B (Fig. 4). Spi55 was a sulfate-reducing bacterium isolated from black rust removed from the borehole seal on Ocean Drilling Program (ODP) Hole 1026B on the ridge flank of the Juan de Fuca Ridge (Nakagawa et al. 2006). Strain Ax17^T^ was also closely related to environmental 16S rRNA gene clones 1026B15 and 1026B_19 from fluid pumped from within the sealed borehole at ODP Hole 1026B (Cowen et al. 2003; Jungbluth et al. 2014). These clones comprised 31% and 5% of the total clones isolated from this site. A similar clone (Dan60_14E) was found in 2 of 40 clones sequenced from production water from a high-temperature oil reservoir in the North Sea (Gittel et al. 2009). The results demonstrate the importance of metabolic versatility of thermophilic autotrophs, including Fe(III) (oxyhydr)oxide mineral reduction, that should be considered when studying CO_2_ fixation in various hot subseafloor environments. Furthermore, the isolation of strain Ax17^T^ using akaganéite and lepidocrocite provides an example of how alternative Fe(III) (oxyhydr)oxide minerals could aid in future efforts to identify other novel iron-reducing strains.

### Description of *Desulfovulcanus* gen. nov

*Desulfovulcanus* (De.sul.fo.vul.ca’nus L. pref. *de*, from; L. neut. n. *sulfur*, sulfur; L. masc. n. *vulcanus*, god of fire; N.L. neut. n. *Desulfovulcanus*, sulfate reducer from a volcano).

Cells are curved to straight rods. Strictly anaerobic. Thermophilic. Chemolithoautotrophic. Able to utilize molecular hydrogen and formate as electron donors and Fe(III) (oxyhydr)oxide minerals, sulfate, sulfite, thiosulfate, and elemental sulfur as electron acceptors. Sodium chloride (NaCl) is absolutely required for growth. Phylogenetically, the genus *Desulfovulcanus* belongs to the family *Desulfonauticaceae*. The type species is *Desulfovulcanus ferrireducens*.

### Description of *Desulfovulcanus ferrireducens* sp. nov

*Desulfovulcanus ferrireducens* (fer.ri.re.du’cens L. neut. n. *ferrum*, iron; *L. pres. part. reducens* bringing back, leading back; N.L. part. adj. *ferrireducens*, iron-reducing).

Cells are Gram negative, curved to straight rods with monopolar flagellation, and approximately 1.5 μm in length and 0.4 μm in width. Growth occurs between 35 and 70 °C, pH 5.0 and 9.0, and 1.5 and 6.0% NaCl. Strictly anaerobic. Chemolithoautotrophic growth occurs with hydrogen and formate as the electron donor and nanophase Fe(III) (oxyhydr)oxide (akaganéite, lepidocrocite, ferrihydrite, hematite, geothite), sulfate, sulfite, thiosulfate, and elemental sulfur as the electron acceptor. No growth is observed when acetate, glucose, tryptone, pyruvate, citrate, succinate, ethanol, glycerol, or yeast extract is used as the electron donor and carbon source. No growth on Fe(III)-citrate, nitrate, or oxygen or purely by fermentation. The genomic DNA G + C content of the type strain is 42.5% based on total genome calculations.

The type strain, Ax17^T^ (= DSM 111878^T^ = ATCC TSD-233^T^) was isolated from low-temperature hydrothermal vent fluid at Axial Seamount, Juan de Fuca Ridge, in the northeastern Pacific Ocean. The GenBank/EMBL/DDBJ accession numbers for the 16S rRNA gene and draft genome sequence of the type strain are MZ048018 and JAGUQP000000000, respectively.
